# Teaching old drugs new tricks

**DOI:** 10.7554/eLife.84702

**Published:** 2022-12-08

**Authors:** Alexandre Faille, Alan J Warren

**Affiliations:** 1 Cambridge Institute for Medical Research, University of Cambridge Cambridge United Kingdom; 2 The Department of Haematology, University of Cambridge Cambridge United Kingdom; 3 Wellcome Trust-Medical Research Council Stem Cell Institute, University of Cambridge Cambridge United Kingdom

**Keywords:** mitochondria, mitoribosome, antibiotics, bacteria, Fe-S cluster, ototoxicity, Human

## Abstract

Understanding the mechanism by which streptomycin binds to the small subunit of the mitoribosome may help researchers design less toxic derivatives of this antibiotic.

**Related research article** Itoh Y, Singh V, Khawaja A, Naschberger A, Nguyen MD, Rorbach J, Amunts A. 2022. Structure of the mitoribosomal small subunit with streptomycin reveals Fe-S clusters and physiological molecules. *eLife* **11**:e77460. doi: 10.7554/eLife.77460.

Most of the classes of antibiotics used today were discovered between the 1940s and 1960s. Over the decades, the widespread use of antibiotics and lack of new drugs has led to a rise in antibiotic resistant bacteria, making it increasingly challenging to treat common infections.

Streptomycin was the first discovered aminoglycoside antibiotic, originally derived from the bacterium *Streptomyces griseus*, and became the primary treatment for tuberculosis and other bacterial infections (Schatz et al., 1944). It eliminates bacteria by irreversibly binding to the small subunit of the machine responsible for producing proteins known as the ribosome, leading to an impaired synthesis of proteins and ultimately the death of the bacterial cell. But streptomycin can also bind to an evolutionarily related ribosome in the mitochondria of humans. This can result in hearing problems known as ototoxicity, which can potentially lead to deafness. Therefore, streptomycin is currently only used as an adjunct treatment if necessary (Waters and Tadi, 2022).

Despite these toxic side effects, the widespread emergence of antibiotic resistance calls for desperate measures and streptomycin is once again in demand to target the bacterium that causes tuberculosis, which has also become resistant to a variety of treatments (Cohen et al., 2020). A better knowledge of the molecular mechanism that enables streptomycin to bind to the human mitochondrial ribosome may help researchers find ways to reduce the ototoxic effects caused by the drug.

Now, in eLife, Alexey Amunts and colleagues from the University of Sweden – including Yuzuru Itoh, Vivek Singh, Anas Khawaja as joint first authors – report new insights into the structure and function of the small ribosomal subunit in human mitochondria that binds to streptomycin (Itoh et al., 2022a). Itoh et al. used a method called single particle electron cryo-microscopy (cryo-EM) to analyse the structure of the mitochondrial ribosome in humans, also known as the mitoribosome. This technique generated a much higher resolution structure of streptomycin bound to the bacterial ribosome than previous X-ray crystallography or previous cryo-EM studies.

The experiments revealed that several physiologically important molecules and clusters of iron and sulphur are integrated into the small subunit of the mitoribosome. This suggests that as the mitoribosome is assembled, these clusters may stabilise interactions between proteins in regions where ribosomal RNA has been deleted over the course of evolution.

The study also indicates a potential regulatory link between the three main metabolic pathways in mitochondria: mitoribosome biogenesis, iron-sulfur assembly, and fatty-acid synthesis. Dysfunctional mitochondria are linked to many diseases, including muscular diseases and Friedreich’s ataxia, and the chemical-level detail provided by Itoh et al. lays the foundation for future efforts to better understand the role of mitochondria in human health and disease (Pirinen et al., 2020; Gomes et al., 2013; Marmolino, 2011).

A highlight of the study by Itoh et al. is the elucidation of the detailed interactions between streptomycin and the mitoribosome (Figure 1). The team showed that the aldehyde group found on the streptose sugar moiety of streptomycin is hydrated to a geminal diol (an organic compound with two hydroxyl groups bound to the same carbon) prior to binding to the ribosome. This hydration enables multiple hydrogen bonding interactions between streptomycin and the phosphate backbone of the ribosomal RNA.

**Figure 1.**
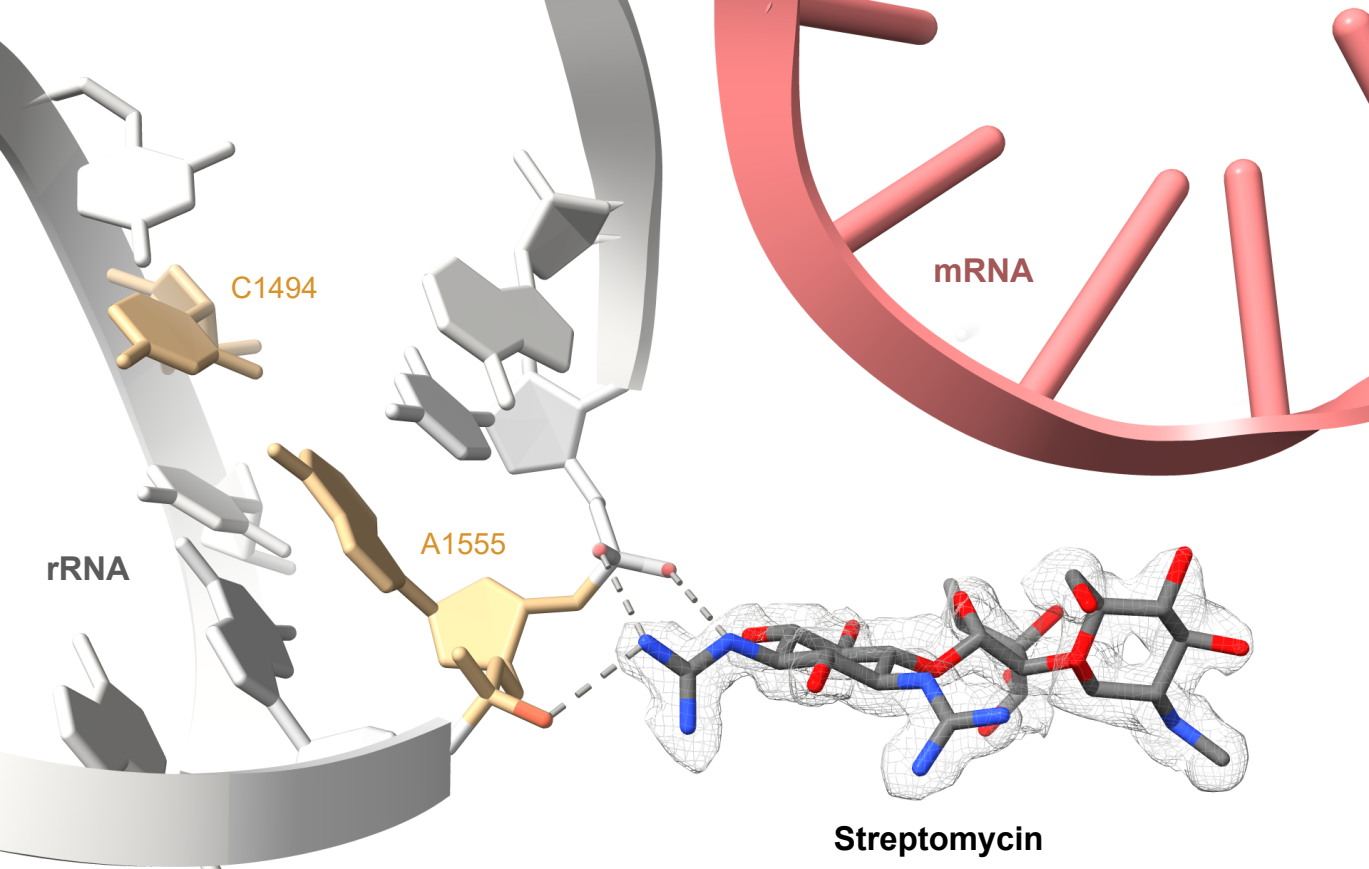
Illustration of how the aminoglycoside antibiotic streptomycin causes ototoxicity. Ototoxicity is a condition where drugs, such as streptomycin, can cause hearing problems and even deafness. It has previously been shown that patients, who are more likely to get ototoxicity, have mutations in the nucleotides located in the mitochondrial ribosome, or mitoribosome. These mutations introduce new RNA base pairings that harden the structure of the small unit of the ribosome (grey). Itoh et al. have shown that streptomycin (red and blue sticks, experimental cryo-EM map is shown as mesh) binds directly to the nucleotides A1555 and C1556 (orange) of the small subunit of the mitoribosome via hydrogen bonds (dashed grey lines), making it even more rigid. This impairs the ribosome’s ability to translate mRNA into proteins (pink ladder mRNA cartoon has been superimposed from the *E. coli* ribosome structure PDB: 7K00 to show where the mRNA is likely positioned in relation to the streptomycin).

The work by Itoh et al. also provides new insights into how streptomycin can cause ototoxicity (Figure 1). Sequencing the mitochondrial DNA of patients suffering from hearing loss has previously revealed frequent mutations in specific nucleotides, which introduce new RNA base pairings that locally rigidify the structure of the mitoribosome (Gao et al., 2017). The researchers found that streptomycin adds to this rigidification by directly binding to one of these nucleotides, which in turn impairs the ribosome’s ability to translate messenger RNA into protein. These important new structural insights explain how hearing loss may be induced or aggravated by streptomycin and may facilitate the design of less toxic aminoglycoside antibiotic derivatives in the future.

Moreover, previous studies have suggested that antibiotics that induce mitochondrial dysfunction could be modified to treat some cancers (Karp and Lyakhovich, 2022). Cancer stem cells depend on oxidative phosphorylation, which can be blocked with certain antibiotics. For example, the antibiotic drug tigecycline can inhibit the translation of mitochondrial proteins and has been proposed as a therapeutic strategy for acute myeloid leukaemia (Skrtić et al., 2011). The exciting study from Itoh et al. may allow other ‘off-target’ antibiotics to be repurposed as cancer therapeutics by harnessing the three-dimensional structure of the drug bound to its biomolecular target for rational drug design. Unravelling the molecular mechanisms that drive the assembly of the mitoribosome is also a fertile area of research that promises to yield additional new targets for cancer therapy beyond the mature mitoribosome (Itoh et al., 2022b).
